# Unique Interactions of the Nuclear Export Receptors TbMex67 and TbMtr2 with Components of the 5S Ribonuclear Particle in Trypanosoma brucei

**DOI:** 10.1128/mSphere.00471-19

**Published:** 2019-08-14

**Authors:** Constance Rink, Noreen Williams

**Affiliations:** a Department of Microbiology and Immunology, University at Buffalo, Buffalo, New York, USA; Carnegie Mellon University

**Keywords:** nuclear export, protein-RNA interactions, protein-protein interactions, ribosome biogenesis

## Abstract

Eukaryotic ribosome biogenesis is a complicated and highly conserved biological process. A critical step in ribosome biogenesis is the translocation of the immature ribosomal subunits from the nucleoplasm, across the nucleopore complex, to the cytoplasm where they undergo final maturation. Many nonribosomal proteins are needed to facilitate export of the ribosomal subunits, and one complex participating in export of the pre-60S in Saccharomyces cerevisiae is the heterodimer Mex67-Mtr2. In Trypanomsoma brucei, the process of ribosome biogenesis differs from the yeast process in key steps and is not yet fully characterized. However, our laboratory has previously identified the trypanosome-specific proteins P34/P37 and has shown that P34/P37 are necessary for the formation of the 5S ribonuclear particle (RNP) and for the nuclear export of the pre-60S subunit. We have also shown that loss of TbMex67 or TbMtr2 leads to aberrant ribosome formation, rRNA processing, and polysome formation in T. brucei. In this study, we characterize the interaction of TbMex67 and TbMtr2 with the components of the 5S RNP (P34/P37, L5 and 5S rRNA) of the 60S subunit. We demonstrate that TbMex67 directly interacts with P34 and L5 proteins as well as 5S rRNA, while TbMtr2 does not. Using protein sequence alignments and structure prediction modeling, we show that TbMex67 lacks the amino acids previously shown to be essential for binding to 5S rRNA in yeast and in general aligns more closely with the human orthologue (NXF1 or TAP). This work suggests that the T. brucei Mex67-Mtr2 binds ribosomal cargo differently from the yeast system.

**IMPORTANCE** Trypanosoma brucei is the causative agent for both African sleeping sickness in humans and nagana in cattle. Ribosome biogenesis in these pathogens requires both conserved and trypanosome-specific proteins to coordinate in a complex pathway. We have previously shown that the trypanosome-speciﬁc proteins P34/P37 are essential to the interaction of the TbNmd3-TbXpoI export complex with the 60S ribosomal subunits, allowing their translocation across the nuclear envelope. Our recent studies show that the trypanosome orthologues of the auxiliary export proteins TbMex67-TbMtr2 are required for ribosome assembly, proper rRNA processing, and polysome formation. Here we show that TbMex67-TbMtr2 interact with members of the 60S ribosomal subunit 5S RNP. Although TbMex67 has a unique structure among the Mex67 orthologues and forms unique interactions with the 5S RNP, particularly with trypanosome-specific P34/P37, it performs a conserved function in ribosome assembly. These unique structures and parasite-specific interactions may provide new therapeutic targets against this important parasite.

## Abstract (precis)

Trypanosoma brucei is the causative agent for both African sleeping sickness in humans and nagana in cattle. Ribosome biogenesis in these pathogens requires both conserved and trypanosome-specific proteins to coordinate in a complex pathway. We have previously shown that the trypanosome-speciﬁc proteins P34/P37 are essential to the interaction of the TbNmd3-TbXpoI export complex with the 60S ribosomal subunits, allowing their translocation across the nuclear envelope. Our recent studies show that the trypanosome orthologues of the auxiliary export proteins TbMex67-TbMtr2 are required for ribosome assembly, proper rRNA processing, and polysome formation. Here we show that TbMex67-TbMtr2 interact with members of the 60S ribosomal subunit 5S RNP. Although TbMex67 has a unique structure among the Mex67 orthologues and forms unique interactions with the 5S RNP, particularly with trypanosome-specific P34/P37, it performs a conserved function in ribosome assembly. These unique structures and parasite-specific interactions may provide new therapeutic targets against this important parasite.

## INTRODUCTION

Trypanosoma brucei is an extracellular protozoan parasite and the causative agent of both human and agricultural African trypanosomiasis (African sleeping sickness and nagana, respectively). Although this parasite has been studied for years, it continues to cause mortality and significant economic impact in sub-Saharan Africa (1). The current chemotherapeutics for the treatment of African sleeping sickness are highly inadequate. The available drugs have harsh side effects, they are difficult to administer in underdeveloped areas, and resistance to these drugs is on the increase (1). These inadequacies underscore the strong need for the discovery of essential biological processes in T. brucei and the identification of parasite-specific interactions that can be exploited for chemotherapeutic intervention.

Ribosome biogenesis is the formation of the large and small ribosomal subunits (60S and 40S, respectively) and, ultimately, the functional ribosome (80S) responsible for protein translation. This biogenesis pathway is a highly conserved and vital process for eukaryotes, including T. brucei. As in other eukaryotes, in T. brucei, ribosome biogenesis begins with the assembly of ribosomal proteins, accessory factors, and four species of rRNA (28S, 18S, 5.8S, and 5S rRNA) within the nucleolus (2). The process of ribosome subunit maturation continues in nucleoplasm where the addition and removal of accessory factors results in specific conformational changes of the ribosomal subunits (particularly the 60S subunit), leading to export competency (2). The pre-60S and pre-40S subunits are then translocated across the nuclear pore by the nuclear export complex, exportin 1 (Xpo1) and the adaptor protein Nmd3 (3).

Even though T. brucei ribosome biogenesis and ribosomal subunit export has not been extensively characterized, these limited studies have already identified striking differences such as the processing of the 25/28S rRNA into six fragments (4). Another unique feature is the presence of the trypanosome-specific RNA binding proteins P34/P37. These essential proteins have been characterized to form a preribosomal complex with the conserved ribosomal protein L5, and 5S rRNA in the nucleoplasm (5–8). In addition, P34/P37 have been shown to associate with both early and late maturation stages of the large ribosomal subunit (60S) (9). Significantly, decreased expression of P34/P37 results in loss of association between the pre-60S particle and the nuclear export complex (T. brucei Xpo1 [TbXpo1] and TbNmd3) (9). Together, these data suggest a potential direct interaction between trypanosome-specific P34/P37 and the nuclear export complex.

In T. brucei, the auxiliary export proteins T. brucei Mex67 (TbMex67) and TbMtr2 have been shown to play a role in mRNA export, and recent work in our laboratory supports its role in ribosomal subunit biogenesis (10). In addition, TbMex67 has been shown to associate with several nuclear pore proteins (11). The structure of TbMex67 is unusual. While it contains a leucine-rich repeat (LRR) domain and an NTF2-like domain as do other Mex67 orthologues, it contains a N-terminal zinc finger motif (CCCH) rather than an RNA recognition motif (RRM) domain and appears to lack or possesses a poorly conserved, ubiquitin-associated (UBA) domain. The zinc finger motif was shown to be essential in mRNA export (10, 11). Despite these structural differences, TbMex67-TbMtr2 retain the conserved function as auxiliary export proteins previously reported for other orthologues. Ultimately, the structural differences of TbMex67-TbMtr2 raise questions concerning the way these proteins associate with ribosomal subunits, particularly the large ribosomal subunit (60S). Adding to this question is the presence of conflicting data in the literature of how eukaryotic Mex67-Mtr2 specifically associates with the pre-60S subunit. It was originally hypothesized that in Saccharomyces cerevisiae, Mex67-Mtr2 association with the pre-60S subunit was through direct interaction with 5S rRNA (12). However, recent data have suggested that ScMex67-ScMtr2 binds to solvent-exposed regions of 28S rRNA and 5.8S rRNA on the 60S subunit (13).

In this work, we ask whether TbMex67-TbMtr2 associates (directly or indirectly) with the large (60S) ribosomal subunit. We have identified new interactions between the auxiliary export receptor proteins TbMex67 and TbMtr2 and the 5S ribonuclear particle (RNP) of the pre-60S ribosomal subunit. We hypothesize that TbMex67 associates with the large ribosomal subunit through direct protein-protein and protein-RNA interactions.

## RESULTS

### Structural analysis of TbMex67 and TbMtr2.

**FIG 1:**
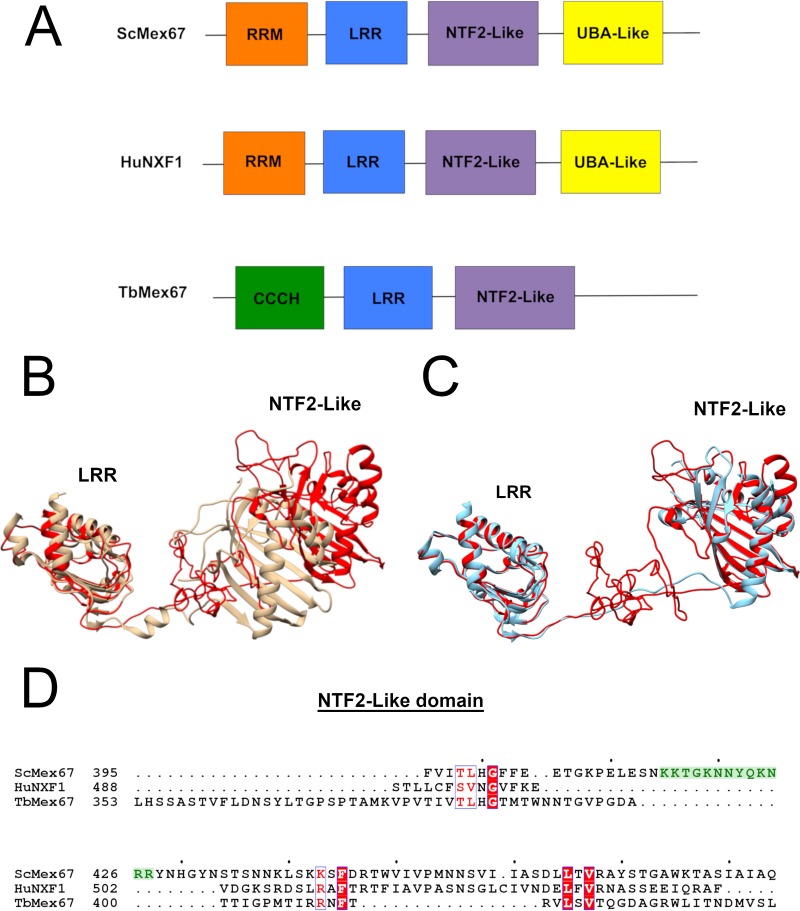
Predicted structure of T. brucei Mex67 (TbMex67) and its similarity to NXF1 (TAP) structure. (A) The structural domain organization of Mex67 orthologues from the N terminus to the C terminus (domains not shown to scale). ScMex67, S. cerevisiae Mex67; HuNXF1, human NXF1. (B) ScMex67 structure lacking the RRM domain (tan) overlaid on the predicted structure of TbMex67 (red) lacking the N-terminal zinc finger. (C) NXF1 (TAP) structure lacking the RRM domain (blue) overlaid on TbMex67 (red) lacking the N-terminal zinc finger. (D) Protein sequence alignment of the NTF2-like domain of Mex67 orthologues. The amino acids important in binding to 5S rRNA in yeast are shown highlighted in green, and conserved amino acids are shown in boxes by white letters on a red background.

Previous work has shown that TbMex67 is structurally unique compared to other Mex67 orthologues (10). Both Saccharomyces cerevisiae and human Mex67 orthologues are comprised of an N-terminal RNA recognition motif (RRM), a leucine-rich repeat (LRR), followed by an NTF2-like domain and a C-terminal ubiquitin-associated (UBA) domain (Fig. 1A) (14–16). The N terminus of TbMex67 is not predicted to contain an RRM structure as found in S. cerevisiae Mex67 (ScMex67) and NXF1 (TAP), the human orthologue of Mex67, but rather possesses a Zn finger motif (10, 14, 16). This Zn finger is followed by an LRR, an NTF2-like domain, and an as-yet-undefined C terminus (Fig. 1A) (10). TbMtr2 is structurally conserved compared to other orthologues, since it is comprised of an NTF2-like domain, as are other Mtr2 orthologues; however, the sequence similarity among all Mtr2 orthologues is low (17).

To further analyze the relative similarity, we compared the full-length predicted structure of TbMex67 to the crystal structures of yeast and human Mex67 orthologues. While the individual LRR and NTF2-like (beta-sheets) domains from all three structures align with each other, the combined structure, including the linker region of TbMex67 is most similar to NXF1 (TAP), the human orthologue (Fig. 1B and C). This is due to the constraints imposed by the LRR and linker regions which in previous work (13) was found to be significant in the formation of the binding surface of ScMex67. The N-terminal Zn finger domain of TbMex67 does not align with either NXF1 (TAP) or ScMex67 but has previously been shown to be essential for TbMex67-Mtr2 function in mRNA export (10, 14–16).

In addition, we performed *in silico* alignments of the primary amino acid sequence, which show that both TbMex67 and NXF1 (TAP) lack amino acids in the NTF2-like domain that are mapped to an extended loop region in ScMex67 (Fig. 1D). This extended loop region (ScMex67 407 to 436 amino acids [aa]), consisting of approximately 30 amino acids, does not appear in the crystal structure of ScMex67 since it is disordered in nature but was shown in earlier work to be the location of the residues critical for 5S rRNA binding (Fig. 1D, highlighted in green) (12). TbMex67 appears to lack this loop region based on Clustal alignment of the NTF2-like domain (Fig. 1D), and previous work has shown that the loop is also absent in the human orthologue NXF1 (TAP) (18). Based on the absence of an extended loop region within the TbMex67 NTF2-like domain and similarity in the predicted tertiary structure, our analysis suggests that TbMex67 is more similar to NXF1 rather than ScMex67 even though ScMex67 is closer on the evolutionary tree to TbMex67 (19). It further suggests that if 5S rRNA binding occurs, it must occur elsewhere in both human and trypanosome orthologues.

**FIG 2:**
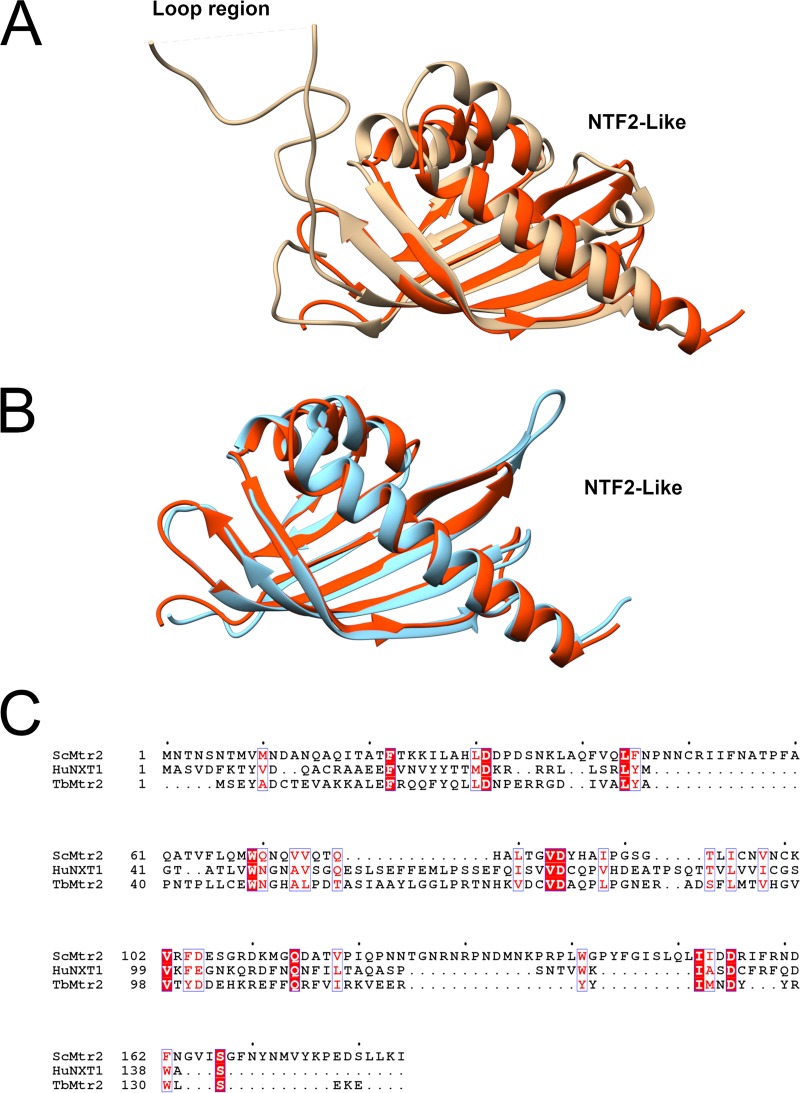
Predicted structure of TbMtr2 and its similarity to human Mtr2 (NXT1) structure. (A) Full-length ScMtr2 structure (tan) overlaid on the predicted structure of TbMtr2 (red). (B) Human NXT1 structure (blue) overlaid on the predicted TbMtr2 structure (red). (C) Sequence alignment of all three full-length Mtr2 proteins, with the amino acids in common indicated by boxes with white letters on a red background.

Similar analysis was performed for Mtr2, and we show that the predicted structure of TbMtr2 is similar to human Mtr2 (NXT1) (Fig. 2). As for other orthologues, TbMtr2 contains an NTF2-like domain (17). A comparison of the NTF2-like domains also shows that the extended loop structure in ScMtr2 (106 to 141 aa) is also not present in TbMtr2 (Fig. 2A and B), as is true for the human orthologue (17). When comparing the amino acid sequences between TbMtr2, ScMtr2, and NXT1, we observed a low number of amino acids in common (Fig. 2C).

### TbMex67 associates with P34/P37 *in vivo*.

Recent work from our laboratory showed that the loss of TbMex67 or TbMtr2 led to a defect in ribosome biogenesis, specifically altered subunit composition, a loss in polysome formation, and aberrant rRNA processing (20). Previous experiments using Ty-tagged TbMex67 or TbMtr2 showed that the protein members of the Tb5S RNP (L5 and P34/P37) associated with TbMex67-Mtr2. We further showed that loss of TbMtr2 and to some extent, TbMex67 impacted levels of these 5S RNP proteins, suggesting an interrelationship (20). These results prompted us to examine these interactions further, specifically in nontagged wild-type cell lines, to determine whether these associations could be validated.

Our laboratory has previously demonstrated that trypanosome-specific proteins P34/P37 associate with the 5S RNP and interact directly with 5S rRNA (8). These proteins are also essential for the export of the pre-60S subunit, and loss of these proteins using RNA interference led to a loss in association of TbXpoI-TbNmd3 with the 60S complex and its subsequent export from the nucleus to the cytoplasm (9). To further understand the role of the T. brucei 5S RNP and its trypanosome-specific components, P34/P37 in pre-60S export, we asked whether P34/P37 associate with TbMex67-TbMtr2.

**FIG 3:**
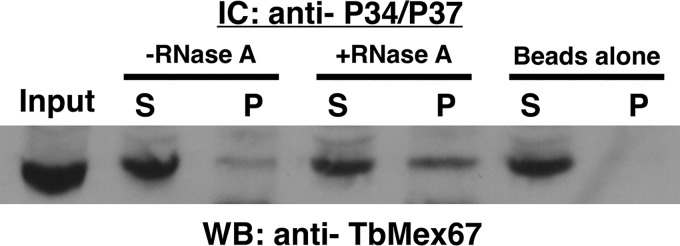
TbMex67 associates with P34/P37 in procyclic cells. Immune capture (IC) was performed with anti-P34/P37 with (+) and without (−) RNase A treatment (whole-cell extract [input, 100 μg], supernatant [S], pellet [P], and beads-alone control) followed by SDS-PAGE and Western blot (WB) analysis probing for TbMex67. The results shown are representative of three biological replicates.

*In vivo* immune capture experiments using procyclic whole-cell extracts were performed using anti-P34/P37 conjugated beads, and Western blot analysis was performed using anti-TbMex67 (Fig. 3). Results from the immune capture consistently showed an average of 0.35 (standard deviation [SD], 0.22) of the relative total amount of TbMex67 present in the pellet fraction. These results demonstrate that TbMex67 associates with P34/P37 *in vivo*. To determine whether RNA influences this association, we added RNase A (an enzyme that cleaves single-stranded RNA) to the whole-cell extract to remove available RNA prior to performing immune capture. Unexpectedly, we observed a modest but consistent increase (0.53 [SD, 0.16] versus 0.35 [SD, 0.22]) in the relative amount of TbMex67 in the pellet compared to the untreated extract pellet. This result indicates that RNA does not enhance the association between P34/P37 and TbMex6,7 but instead, it may be inhibitory. However, due to the nature of RNase treatment, there is the possibility of embedded RNAs, untouched by treatment, which could be aiding in the protein-protein interactions.

### TbMex67 directly interacts with P34, and 5S rRNA does not significantly influence this interaction.

In order to further characterize the nature of the association between P34/P37 and TbMex67, we performed immune capture experiments with recombinant His-tagged TbMex67 and P34. Specifically, we were interested in determining whether the association between these proteins was a direct protein-protein interaction. For these experiments, we chose to examine only the P34 protein, since P34 and P37 are functionally identical (8, 21).

**FIG 4:**
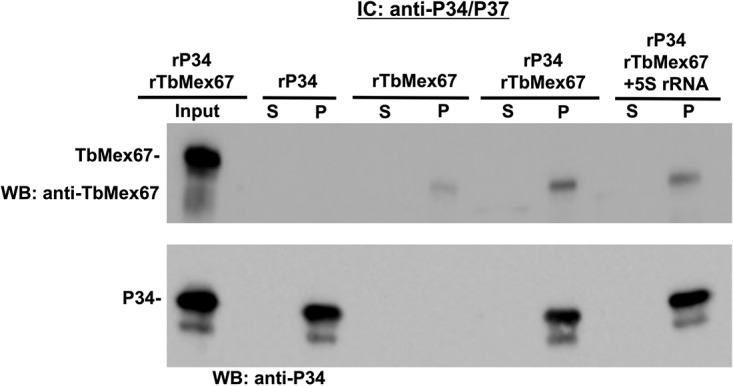
TbMex67 directly associates with P34, and 5S rRNA does not influence this interaction. Immune capture (IC) was performed with anti-P34/P37 using equal molar amounts of each protein and 5S rRNA as indicated in the figure. Input proteins (as indicated), supernatant (S), and pellet (P) were subjected to SDS-PAGE and Western blot (WB) analysis probing for TbMex67 and anti-P34. The results shown are representative of three biological replicates. rP34, recombinant P34; rTbMex67, recombinant TbMex67.

The recombinant immune capture experiments were performed using anti-P34/P37 conjugated beads followed by Western blot analysis using anti-TbMex67 or anti-P34 antibodies (Fig. 4). In control experiments, we observed that P34 was captured by the P34/P37 antibody-specific beads (positive control), whereas TbMex67 was not (negative control). When both proteins were incubated together at an equal molar ratio, both TbMex67 and P34 were found in the pellet, demonstrating a direct interaction between them.

In addition, we wanted to determine whether 5S rRNA could influence (either enhancing or disrupting) the interaction between P34 and TbMex67. Therefore, we performed the same recombinant immune capture experiment with the addition of *in vitro*-transcribed 5S rRNA. Interestingly, we observed only a modest change in the direct association between P34 and TbMex67 in the presence of 5S rRNA (0.94 [SD, 0.17]) (Fig. 4). These results suggest that additional ribosomal RNAs besides 5S rRNA could be influencing the interaction observed *in vivo*. Overall, these results show previously uncharacterized interactions and further demonstrate the uniqueness of TbMex67 and trypanosome-specific proteins P34/P37.

### TbMex67 associates with conserved ribosomal protein L5 *in vivo*.

With the discovery of the P34/P37 association with TbMex67 in T. brucei extracts, we next asked whether TbMex67 might also associate with ribosomal protein L5, another component of the 5S RNP. From previous findings, we know that L5 forms a preribosomal complex with P34/P37 and 5S rRNA in T. brucei (6, 7). We have shown that within this complex, L5 directly binds to and has a strong affinity for 5S rRNA; it also has direct interactions with P34/P37 (6, 7). These data led us to believe that if TbMex67 associates with P34/P37, L5 may be in close proximity to or associated with TbMex67.

**FIG 5:**
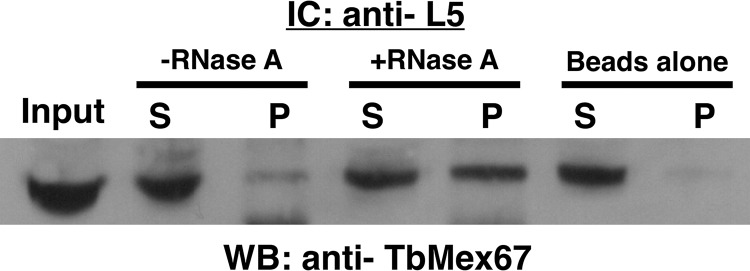
TbMex67 associates with L5 in procyclic cells. Immune capture was performed with anti-L5 with and without RNase A treatment (whole-cell extract [input, 100 μg], supernatant [S], pellet [P], and beads-alone control), and samples were subjected to SDS-PAGE and Western blot analysis probing for TbMex67. The results shown are representative of three biological replicates.

To determine whether L5 associates with TbMex67, we performed immune capture experiments with anti-L5 conjugated beads and Western blot analysis using anti-TbMex67 (Fig. 5). In procyclic whole-cell extracts, we found a consistent fraction of the total amount of TbMex67 (0.43 [SD, 0.21]) associated with L5, a similar result to that previously observed with P34/P37. When extracts were treated with RNase A prior to immune capture, we observed an increased amount (0.80 [SD, 0.20] versus 0.43 [SD, 0.21]) of TbMex67 in the pellet compared to the untreated extract. This suggests that RNA decreases the *in vivo* interaction between L5 and TbMex67, similar to what we observed for P34 and TbMex67.

### TbMex67 directly interacts with L5, and 5S rRNA does not significantly influence this interaction.

**FIG 6:**
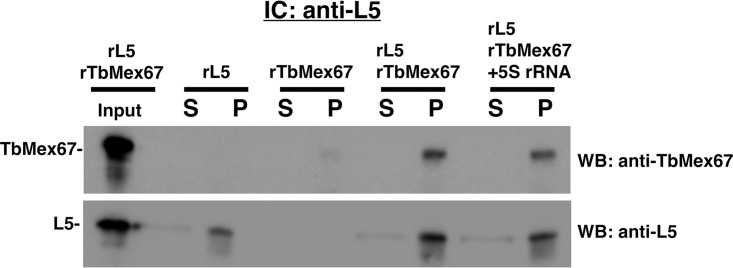
TbMex67 directly associates with L5, and 5S rRNA does not influence this interaction. Immune capture was performed with anti-L5 using equal molar amounts of each protein and 5S rRNA as indicated in the figure. Input proteins (as indicated), supernatant (S), and pellet (P) were subjected to SDS-PAGE and Western blot analysis probing for TbMex67 and anti-His. The results shown are representative of three biological replicates. rL5, recombinant L5.

To further examine the association between L5 and TbMex67 and, specifically, to determine whether the association is a direct protein-protein interaction, we performed *in vitro* recombinant immune capture experiments. These experiments were performed with anti-L5 conjugated beads, and the specific interactions were probed using either anti-TbMex67 or anti-L5 antibodies. We confirmed that recombinant L5 was captured by our antibody-specific beads (Fig. 6). When both proteins were incubated together at an equal molar ratio, we observed both TbMex67 and L5 in the pellet, demonstrating a direct interaction. The addition of 5S rRNA to this reaction mixture showed a slight change in the direct association between L5 and TbMex67 (0.94 [SD, 0.28]) (Fig. 6). These data show that the presence of 5S rRNA does not significantly disrupt the direct interaction between these two proteins and is similar to the previous *in vitro* TbMex67-P34 data.

### TbMtr2 does not directly interact with P34 or L5.

Previous *in vivo* coimmunoprecipitation has shown that TbMex67 and TbMtr2 associate with each other (10). However, it has not been determined whether TbMtr2 participates in additional protein-protein interactions. In the work above, we demonstrated that the TbMex67 protein alone can participate in direct interactions with P34 and L5 *in vitro*.

**FIG 7:**
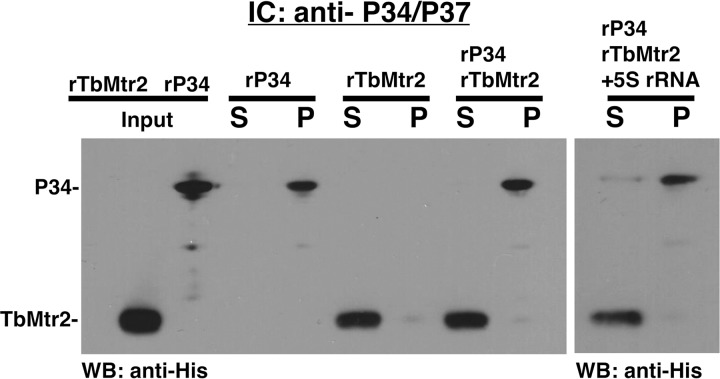
TbMtr2 does not directly associate with P34 in the presence or absence of 5S rRNA. Immune capture was performed with anti-P34/P37 using equal molar amounts of each protein and 5S rRNA as indicated in the figure. Input proteins (as indicated), supernatant (S), and pellet (P) were subjected to SDS-PAGE and Western blot analysis using anti-His. The results shown are representative of three biological replicates.

To address the question of whether TbMtr2 interacts with P34 or L5, we used *in vitro* recombinant immune capture experiments. These experiments were performed using either anti-P34/P37 or anti-L5 conjugated beads, and Western blot analysis of specific interactions was probed with an anti-His antibody. We first examined the potential interaction between P34 and TbMtr2 (Fig. 7). We observed that when incubated together at an equal molar ratio, P34 was the only protein present in the pellet fraction, thus indicating that TbMtr2 and P34 do not directly interact. Further, *in vitro*-transcribed 5S rRNA did not facilitate an association between these proteins.

**FIG 8:**
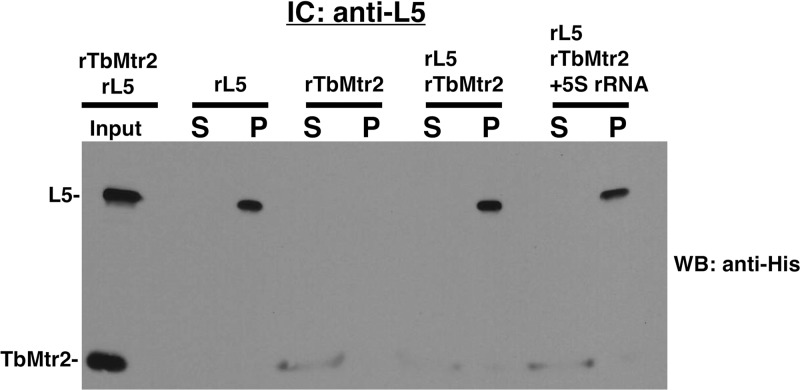
TbMtr2 does not directly associate with L5 in the presence or absence of 5S rRNA. Immune capture was performed with anti-L5 using equal molar amounts of each protein and 5S rRNA. Input proteins (as indicated), supernatant (S), and pellet (P) were subjected to SDS-PAGE and Western blot analysis using anti-His. The results shown are representative of three biological replicates.

We then examined the potential interaction between L5 and TbMtr2, finding a similar result. When recombinant TbMtr2 and L5 were incubated together in an equal molar ratio, L5 was the only protein present in the pellet (Fig. 8), showing that TbMtr2 and L5 do not directly interact *in vitro*. The addition of 5S rRNA to this reaction mixture did not facilitate a protein-protein association. These experiments suggest that TbMtr2 does not directly interact with the other protein components of the 5S RNP.

### TbMex67-TbMtr2 directly binds 5S rRNA through TbMex67.

It has been hypothesized that in yeast, ScMex67-ScMtr2 associates with the 60S ribosomal subunit by directly binding to 5S rRNA (12). Here we have shown that TbMex67 interacts with protein components of the preribosomal complex, the 5S RNP. We have further shown that 5S rRNA alters the interaction of TbMex67 and one of the 5S RNP proteins, P34. In light of the data presented above indicating an association with other preribosomal complex proteins, we wished to determine whether TbMex67 or TbMex67-TbMtr2 complex binds to 5S rRNA directly or whether its impact was indirect.

**FIG 9:**
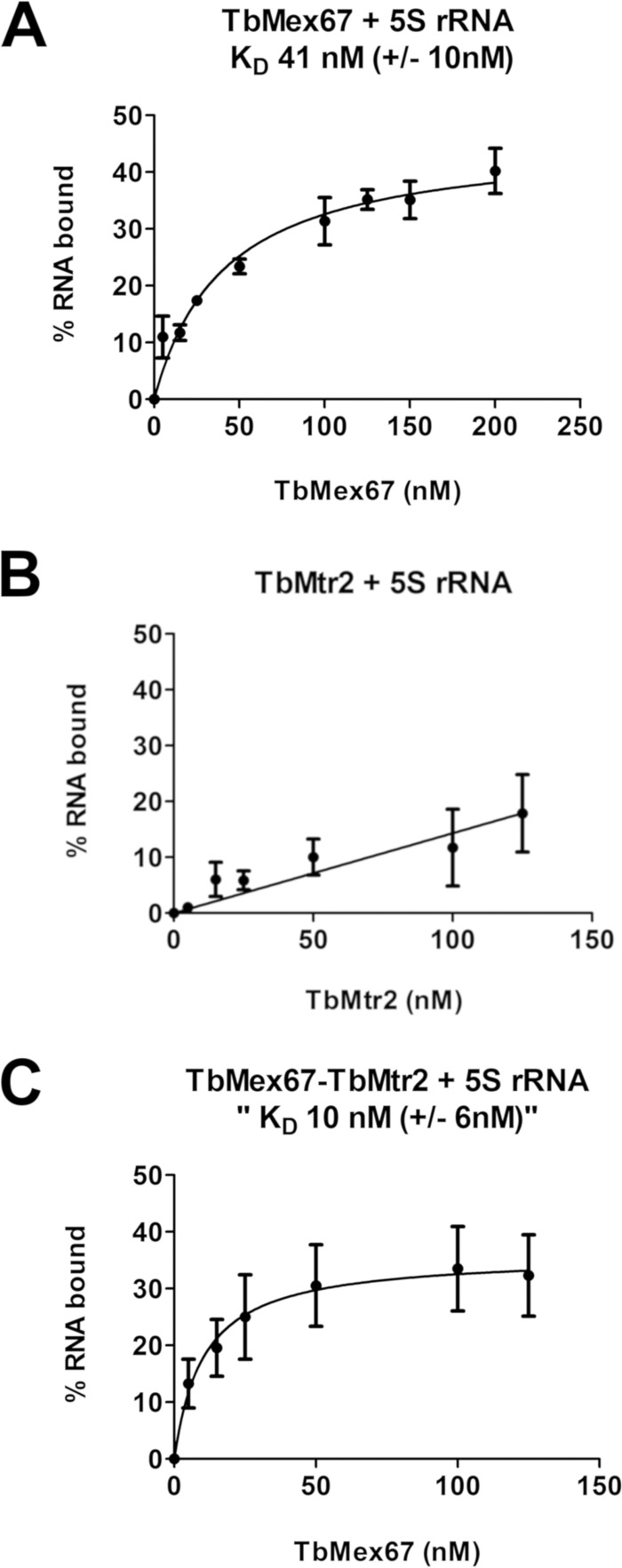
TbMex67-TbMtr2 directly binds to 5S rRNA through TbMex67. (A to C) Purified recombinant His fusion TbMex67 and TbMtr2 were incubated in increasing concentrations individually (A and B, respectively) or together with radiolabeled 5S rRNA in RNA binding buffer containing BSA (C). Bound complexes (nitrocellulose membrane) and unbound rRNA (Nytran membrane) of three biological replicates were visualized by Typhoon imager and quantified using Quantity One.

To address this question, we utilized the double-membrane filter binding assay. We observed that in our filter binding experiments, TbMex67 alone was able to bind 5S rRNA directly with a binding affinity (*K_D_*) of 41 ± 10 nM (Fig. 9A). Interestingly, we found that TbMtr2 alone was unable to bind to 5S rRNA with specificity (Fig. 9B). However, when both proteins were preincubated together, TbMex67 and TbMtr2 were able to bind to 5S rRNA with enhanced binding. When the binding curve is represented as the total amount of TbMex67 in the combined reaction, TbMex67 binds to 5S rRNA with a *K_D_* of 10 ± 6 nM (Fig. 9C). Thus, the presence of TbMtr2 changed the binding of TbMex67 to 5S rRNA. Overall, these data show that TbMex67 alone binds directly to 5S rRNA, although it binds with a higher affinity when in a complex with TbMtr2.

## DISCUSSION

The work presented here underscores the significance of a number of unique features of the trypanosome Mex67-Mtr2 complex and further identifies and characterizes the interaction between that complex and the components of the trypanosome 5S RNP, part of the 60S ribosomal subunit. We have previously shown that the trypanosome 5S RNP possesses a number of unique characteristics, including significant differences in the generally conserved L5 protein and the presence of the trypanosome-specific proteins P34/P37 (6–8). We have also shown that loss of the P34/P37 proteins leads to a loss in association of the 60S ribosomal subunit with the TbNmd3-TbXpoI complex and subsequent loss in the export of the 60S subunit to the cytoplasm (9). Most recently, we have shown that loss of the TbMex67 or TbMtr2 leads to a disruption of ribosome biogenesis, including the formation of aberrant subunits, incomplete processing of rRNAs, and loss of formation of polysomes (20). Here, we show that the auxiliary export complex TbMex67-TbMtr2 interacts with the Tb5S RNP components of the 60S complex. We have also demonstrated in this study that TbMex67-TbMtr2 is involved in unique interactions with the components of the trypanosome 5S RNP (P34/P37, L5, and 5S rRNA) *in vivo* and *in vitro*.

We first performed immunoprecipitation using wild-type procyclic (nontagged) cell lines to confirm our previous results from endogenous tagged procyclic cell lines (20). We showed that TbMex67 associates *in vivo* with both P34/P37 and ribosomal protein L5. Since a similar result was observed for both P34/P37 and L5, we hypothesize that this could be due to the presence of both P34/P37 and L5 in the 5S RNP complex. From our *in vivo* experiments, we found less than half (0.35 to 0.43) of TbMex67 associating with P34/P37 and L5, which is not surprising since Mex67 is proposed to associate with other cargo (22). It should be noted that the proportion of Mex67 protein for each cargo has yet to be determined in other systems. Further experiments *in vitro* showed that the protein-protein associations were direct interactions between TbMex67 and P34/P37 as well as between TbMex67 and L5, suggesting that TbMex67 associates with the protein components of the pre-60S subunit.

Previous data have shown that the direct interaction between ScMex67-Mtr2 and the pre-60S subunit occurs through component rRNA (12, 13). However, ScMex67-ScMtr2 has been shown to interact with many other proteins, including mRNA machinery proteins (TREX), and nuclear pore proteins (Nup84 complex) (23, 24). Specifically, it is hypothesized that in the human system, the interaction between NXF1-NXT1 (orthologues of Mex67-Mtr2) and cellular mRNA in the export process is mediated through the protein-protein interactions of the TREX adaptor proteins (25, 26). These protein-protein interactions are critical for the recognition and binding of NXF1-NXT1 to mRNA because of its apparent nonspecific binding activity (25, 26). Our work here demonstrates a direct Mex67-Mtr2 interaction with the protein components of the 60S ribosomal subunits, and this study adds to our previous understanding obtained from other organisms of how Mex67-Mtr2 participate in nuclear export.

Next we examined whether rRNA played a role in the protein-protein interactions we had observed. Interestingly, we found that RNA present in cell extracts impacts the interactions of TbMex67 with P34/P37 and L5 by decreasing their protein-protein interaction or perhaps by competing for their interactions. *In vitro* data showed that 5S rRNA is not likely responsible for the majority of this change in the interaction between TbMex67 and P34/P37 or L5.

As indicated above, the decrease in the association between TbMex67 and P34/P37 or L5 in the presence of RNA *in vivo* does not seem to be mediated by 5S rRNA based on *in vitro* data. We hypothesize that 25S rRNA or 5.8S rRNA may be influencing these interactions. Recent work on the ScMex67-Mtr2 suggests that 25S and 5.8S may play a role in the interaction of that complex with the 60S subunit (13). We were able to map the specific solvent-exposed regions found to associate with ScMex67-Mtr2 (data not shown) onto the T. brucei cryo-electron microscopy (cryo-EM) structure of the mature 60S subunit, suggesting that they are available for interaction.

To better understand the role of 5S rRNA in these interactions, we examined the binding of 5S rRNA directly with TbMex67 and TbMtr2. Filter binding assays demonstrated that the TbMex67-TbMtr2 complex directly associates with 5S rRNA through TbMex67. TbMex67 associates with 5S rRNA in a saturable manner with a *K_D_* of 41 nM. TbMtr2 was not able to bind 5S rRNA with specificity. However, the addition of TbMtr2 to the assay with TbMex67 and 5S rRNA increased the binding affinity (*K_D_* of 10 nM), suggesting that although it does not directly interact with 5S rRNA, it contributes to binding by the TbMex67-TbMtr2 complex.

TbMex67 was initially reported to lack the NTF2-like domain, which has been linked with RNA binding; however, more recent structural analysis of TbMex67 has identified the presence of an NTF2-like domain (10, 11). *In silico* analysis of the NTF2-like domain of TbMex67 shows that it lacks the amino acids previously identified in ScMex67 to be required for binding to 5S rRNA (Fig. 1C). In yeast, mutation of six positively charged amino acids located within an extended loop structure of the NTF2-like domain of ScMex67 caused a loss of binding to 5S rRNA (12). This region is not present in the trypanosome protein or in the human NXF1 (TAP) protein, and the absence of these residues in TbMex67 does not lead to a decreased capacity of binding to 5S rRNA based on our analysis. This leads us to ask how TbMex67 binds to 5S rRNA. In human NXF1 (TAP) binding to mRNA, cargo was shown to utilize multiple domains, including the end terminal RRM motif, LRR, and the NTF2-like domain (27). The interaction of TbMex67-TbMtr2 with 5S rRNA may be structurally mediated (creation of an RNA binding surface/pocket) rather than through specific sequences (28) and may require multiple domains. Alternatively, TbMex67-TbMtr2 could bind to 5S rRNA through the zinc finger motif located at the N terminus of TbMex67, which has previously been characterized to play a significant role in TbMex67 function in mRNA export (10). The predicted structure of TbMex67 suggests that the zinc finger motif is solvent exposed and therefore would be available for interaction with RNA (data not shown), and future experiments will examine these possibilities.

Our data also suggest that TbMex67 may also function independently from TbMtr2, since we showed that it binds directly with 5S rRNA without the need for additional factors. The human orthologue of Mex67, NXF1 (TAP), has previously been observed to participate in mRNA export independently from dimerization with Mtr2 (29). While TbMtr2 alone was unable to bind to 5S rRNA, we did observe a significant impact on the binding of TbMex67 to 5S rRNA when TbMtr2 was added. The observed change in the binding curve and increased affinity lead us to hypothesize that the presence of TbMtr2 impacts the structure of TbMex67 and/or the structure of 5S rRNA, resulting in an increased binding affinity as a complex. These results may support the involvement of multiple structural components, which form an enhanced binding surface as described above (16). Future experiments will examine whether this hypothesis is correct.

The results from these experiments provide a better understanding of the interaction between Mex67-Mtr2 and their associated cargo and may apply to some other orthologues (e.g., human). However, some features are clearly unique to trypanosomes, since the TbMex67-TbMtr2 complex has trypanosome-specific structural (presence of the Zn finger motif) and functional (interaction with P34/P37) features. These and other previously described trypanosome-specific features of ribosome biogenesis will be the focus of future studies to develop targets for chemotherapeutics.

## MATERIALS AND METHODS

### Protein modeling and sequence alignment.

Trypanosoma brucei Mex67 (TbMex67) (TriTrypDB accession no. Tb427tmp.22.0004) and TbMtr2 (TriTrypDB accession no. Tb427.07.5760) protein sequences were submitted to the I-TASSER server (30) to predict its structure. Following the structure prediction, TbMex67 or TbMtr2 was modeled and overlaid with the known structures of Saccharomyces cerevisiae Mex67 (ScMex67) or ScMtr2 (RCSB PBD accession no. 4WWU) and NXF1 (TAP) or NXT1 (RCSB PBD accession no. 4WYK) using UCSF Chimera software package (31). In addition, the protein sequences of TbMex67, ScMex67 (UniProt accession no. Q99257), NXF1 (UniProt accession no. Q9UBU9), TbMtr2, ScMtr2 (UniProt accession no. P34232), and NXT1 (UniProt accession no. Q9UKK6) were aligned using Clustal Omega (32) and the alignments were presented using the EsPript3 server (33).

### Cell culture.

Procyclic T. brucei *brucei* strain 427 was grown in Cunningham’s medium supplemented with 10% fetal calf serum (9).

### *In vivo* immune capture experiments.

Immune capture experiments were performed using antibodies specific to P34/P37 or L5. Wild-type procyclic cells (427 strain) were hypotonically lysed creating a whole-cell extract. One hundred micrograms of extract was incubated with antibody cross-linked protein A Dynabeads (Invitrogen) in phosphate-buffered saline containing 10 mg/ml bovine serum albumin (BSA), with and without RNase A (50 μg) (Thermo Fisher Scientific) for 1 h at 4°C. After incubation, supernatants were collected, and 2% deoxycholate (DOC)−trichloroacetic acid (TCA) was added and incubated at 4°C overnight. After sedimentation, the pellets were washed once with acetone and then suspended in SDS-PAGE sample buffer. The beads were washed three times with phosphate-buffered saline containing 0.05% Tween 20 and were then heated at 70°C for 10 min with SDS-PAGE sample buffer to elute off bound proteins. Cell extracts (input, 100 μg), supernatants, and pellets were subjected to 10% SDS-PAGE and Western blot analysis to detect any interaction with TbMex67 (1:2,000). Individual blots were analyzed using Quantity One software, and for each analysis, input was set at 1.0, the mean was calculated for the pellet fraction, and standard deviations (SDs) were calculated for three biological replicates.

### Antibodies.

The antibodies used in this study were as follows: P34/P37 antibody (1:2,000; Bethyl Laboratories), TbMex67 antibody (1:2,000; a gift from Mark Carrington, Cambridge), L5 antibody (1:2,000; Bethyl Laboratories), Ty tag-specific antibody (1:2,000; Thermo Fisher Scientific), and His antibody (1:1000; Invitrogen).

### Protein purification.

Recombinant His-tagged P34 (TriTrypDB accession no. Tb427tmp.01.5570), TbMex67 (TriTrypDB accession no. Tb427tmp.22.0004), TbMtr2 (TriTrypDB accession no. Tb427.07.5760), and L5 (TriTryp accession no. Tb427tmp.244.2740) (pTrcHis; Invitrogen) were expressed separately in Escherichia coli cells (TOP10; Invitrogen). Cultures were grown to an optical density (OD) of 0.6 and induced with 1 mM isopropyl-β-d-thiogalactopyranoside (IPTG) for 3 to 4 h at 37°C. All buffers used in purification included protease inhibitor cocktail tablet(s) (Roche) and were degassed. Cells were suspended in lysis buffer (50 mM NaH_2_PO_4_, 300 mM NaCl) containing lysozyme (1 mg/ml) on ice for 1 h, followed by pulse sonication for 1 min (30 s on, 45 s off, intervals). Lysates were sedimented for 20 min at 10,000 × *g*, and cleared lysate was poured over a lysis buffer-equilibrated nickel-nitrilotriacetic acid (NTA) column. The column was washed twice with wash buffer (50 mM NaH_2_PO_4_, 300 mM NaCl, 20 mM imidazole, 0.1% Tween 20), tagged proteins were eluted off the column with elution buffer (50 mM NaH_2_PO_4_, 300 mM NaCl, 250 mM imidazole), and 10 fractions were collected. Eluates were subjected to 10% sodium dodecyl sulfate-polyacrylamide gel electrophoresis (SDS-PAGE), Coomassie staining, Western blot analysis, and Bradford protein analysis. Eluted fractions were pooled, flash frozen in liquid nitrogen, and stored at –80°C.

### Filter binding experiments.

Recombinant His fusion proteins of TbMex67 and TbMtr2 were expressed in E. coli Top10 cells and purified by affinity nickel chromatography as described above. Radiolabeled 5S rRNA was *in vitro* transcribed using PCR-generated DNA template containing the 5S gene, ribosomal nucleoside triphosphates (NTPs), alpha-UTP (Perkin Elmer), and T3 RNA polymerase (Thermo Fisher Scientific). Unincorporated NTPs were removed from the reaction using a NucAway column (Invitrogen), and the radiolabeled RNA product was electrophoresed on a 5 to 10% TBE (Tris-borate-EDTA) gel to confirm a full-length product. Radiolabeled 5S rRNA was heated to 55°C for 10 min and then cooled on ice, followed by dilution into RNA binding buffer (pH 7.4) (10 mM Tris-HCl, 100 mM NaCl, 1 mM EDTA) containing BSA (100 μg/ml) at a final concentration of 0.05 nM. Then TbMex67 and TbMtr2 were added individually (or combined) in increasing concentrations (0 to 200 nM) to the diluted radiolabeled 5S rRNA, and the mixtures were incubated for 30 min at room temperature. After incubation, the protein-RNA mixture was added to the 96-well filter binding apparatus (Bio-Rad), and a vacuum was applied. Both the nitrocellulose membrane (containing bound complexes) and Nytran membrane (containing unbound radiolabeled RNA) were visualized using a Typhoon imager. Individual dot densities of membrane images from three biological replicates were analyzed using Quantity One software, and relative binding constant graphs were generated using GraphPad Prism 5 software.

### Recombinant immune capture experiments.

Recombinant immune capture experiments were performed using antibodies specific to P34/P37 or L5. An equal molar ratio of the indicated recombinant protein(s) was incubated for 1 h at 4°C with antibody cross-linked protein A Dynabeads (Invitrogen) in phosphate-buffered saline containing 100 μg/ml BSA. For experiments that included *in vitro*-transcribed 5S rRNA, RNA was added before proteins at an equal molar ratio. After incubation, supernatants were collected and TCA was precipitated as described above. Beads were washed three times with phosphate-buffered saline with 0.05% Tween 20 and eluted at 70°C with SDS-PAGE sample buffer. Samples were then subjected to SDS-PAGE and Western blot analysis using protein-specific antibodies (P34/P37, L5, TbMex67) or anti-His (Invitrogen). The results shown are representative of the three biological replicates. Densitometric quantifications were obtained using Image Lab (Bio-Rad), and the ratio of the amount of TbMex67 found in the pellet (comparing with and without 5S rRNA) was calculated with standard deviation (SD) for TbMex67-P34/P37 and TbMex67-L5 experiments.

### Western blot analysis.

After SDS-PAGE using NuPAGE 10% Novex Bis-Tris gels (Life Technologies), the proteins were transferred to nitrocellulose membranes (Qiagen) and blocked in StartingBlock PBS blocking buffer (Thermo Fisher Scientific). The membranes were incubated with appropriate primary antibody overnight, followed by four 10-min washes with Tris-buffered saline with Tween 20 and then incubation with appropriate horseradish peroxidase-conjugated secondary antibody for 1 h. Proteins were detected using either SuperSignal West Pico chemiluminescence substrate or SuperSignal West Femto maximum sensitive substrate (Pierce).
